# Towards Mechanism-Based Treatments for Fragile X Syndrome

**DOI:** 10.3390/brainsci9080202

**Published:** 2019-08-16

**Authors:** Daman Kumari, Inbal Gazy

**Affiliations:** Section on Gene Structure and Disease, Laboratory of Cell and Molecular Biology, National Institute of Diabetes, Digestive and Kidney Diseases, National Institutes of Health, Bethesda, MD 20892, USA

Fragile X syndrome (FXS) is the most common heritable form of intellectual disability, as well as the most common known monogenic cause of autism spectrum disorder (ASD), affecting 1 in 4000–8000 people worldwide. Almost 30 years ago, in 1991, the causative mutation for FXS was identified to be a CGG-repeat expansion in a gene named *Fragile X Mental Retardation-1* (*FMR1*) located on the X chromosome [1,2]. At that time, it also became clear that methylation of a CpG island proximal to the repeats results in loss of gene expression and disease pathology [3,4,5,6,7]. While many decades have elapsed since these early discoveries, the underlying mechanisms involved in the expansion mutation, as well as the resulting gene silencing, still remain elusive.

Soon after the identification of *FMR1*, a knockout mouse model was developed to better understand the role of *FMR1* and its protein product, FMRP, in FXS [8]. Work from this mouse model, as well as other models, implicated FMRP activity in brain development, synaptic plasticity and neuronal transmission circuits [9,10]. This body of work led to clinical trials attempting to correct the deficient pathways in the brain of FXS patients [11]. However, all these clinical trials, although based on successful preclinical studies, did not show beneficial effects in FXS patients. Thus, currently there is no cure or effective treatment for FXS, and all the available interventions are focused on managing patient symptoms.

Confronted with the gaps in knowledge and lack of treatments for FXS patients, the scientific community decided to revisit the approaches and practices used, implementing changes both in the preclinical and the clinical arenas. This special issue addresses some of the changes that are being made in the field towards finding effective treatments for FXS.

As reviewed by Kumari et al. [12], there are two major avenues currently being pursued for development of effective treatments: (1) Targeting pathways altered in the absence of FMRP in the brain, and (2) Restoring FMRP expression. The fact that clinical trials focused on the restoration of altered pathways have not shown much promise to date, together with the development of new techniques and approaches in the biomedical field, has led many scientific groups to revisit the restoration of FMRP as a potential treatment approach for FXS. Several strategies are currently being investigated as potential ways to restore FMRP expression in patients. One such strategy is gene therapy, wherein, FMRP could be produced from an exogenous DNA introduced into patients’ cells. Hampson et al. [13] discuss this approach and the current work using viral vectors to introduce the FMRP coding sequence into patients. While studies using adeno-associated viral vector FMRP therapy in the *Fmr1* KO mouse model are showing promising results, additional work needs to be done in this area before such treatments can be considered for clinical trials.

Given that the loss of *FMR1* expression leads to the absence of FMRP in the majority of FXS patients, and that epigenetic silencing is reversible, another approach for restoring FMRP would be to reactivate the silenced *FMR1* gene. Understanding the molecular mechanism underlying *FMR1* silencing in FXS is key for the better identification of targets for its reactivation. In this regard mouse models cannot be used as they fail to recapitulate the repeat mediated *FMR1* gene silencing seen in humans. Thus, human cell lines are currently the most widely used model for such research. In this special issue, Abu Diab et al. [14] describe the use of pluripotent stem cells as a model for investigating both the timing and mechanism of gene silencing. They also describe how such models have been used to understand the mechanism of repeat expansion. Kumari et al. [12] expand further on the use of cultured cells as a model system to investigate gene silencing. The authors describe what has been learnt from these models about the pathways involved in silencing and how this knowledge can enable us to develop mechanism-targeted drugs for gene reactivation. They further describe how unbiased screening in cell culture models can be used as an alternative to identify small molecules that target silencing pathways and could potentially restore *FMR1* expression in FXS patients.

The third strategy discussed in this special issue for the restoration of FMRP expression in patients utilizes the CRISPR-Cas9 gene-editing technology. Yrigollen et al. [15] discuss the use of CRISPR-Cas9 as an alternative method for the reactivation of the *FMR1* gene via two different approaches: the use of Cas9 to (1) facilitate the recruitment of either DNA demethylases or transcriptional activators to the *FMR1* gene, or (2) delete the CGG repeats, which will hopefully lead to the loss of methylation. While such approaches are very appealing, they have many limitations such as their efficacy, off-target effects and efficiency of delivery. Thus, as with the previously described approaches, further study is required before they can be considered for therapeutic uses.

Another avenue for therapeutic targeting is to reduce, if not eliminate, the pathological expansions of the CGG repeats. To identify such targets, understanding the mechanism underlying the CGG-repeat expansion mutation is crucial. Moreover, better understanding of the expansion mechanism can also enable clinicians to better assess disease risk in patients who display high variability in the extent of expansions as well as the penetrance and manifestation of disease. Zhao et al. [16] address the current knowledge from an *Fmr1* KI mouse model, and the potential implications for humans. Saré et al. [17] present data on a different mouse model, investigating the function of FXR2P, an FMRP paralog, as a possible modulator of FXS phenotypes. Applying the lessons learnt from different mouse models to develop research in humans has the potential to increase our understanding of disease risk and repeat instability, develop better diagnostic tools for use in the clinic, identify outcome measures for clinical trials and discover new targets for drugs.

Regardless of the therapeutic approach, when moving from preclinical studies to clinical trials, objective, measurable and reliable molecular biomarkers that differentiate healthy controls from patients are necessary for evaluating the efficacy of the drugs used in the clinical trials. The failure of clinical trials in finding drugs that can be used specifically for FXS has drawn attention to the need for finding better outcome measures in FXS clinical trials. Such need is highlighted by the fact that placebo response in FXS clinical trials is strong, which can result in difficulties in assessing positive responses. Zafarullah et al. [18] list in their review the currently known candidate molecular biomarkers, and Pal et al. [19] focus on one such measure, protein synthesis, as a potential biomarker. While there are a few potential candidates, to date none of these biomarkers have been shown to be robust, reliable or accurate, or can be measured in an accessible tissue (such as blood). Therefore, there is still an urgent need to find appropriate biomarkers for the assessment of drug response in FXS clinical trials in parallel to advancing novel drug discovery.

Fragile X syndrome is a multi-symptomatic disorder and in addition to intellectual disability and ASD, there are many associated behavioral symptoms such as anxiety, depression and others. As discussed above, scientists are trying to find drugs that will be able to cure FXS, yet, until then, treatments for FXS are focused on ameliorating specific symptom/s of patients. However, what symptom/s should be the focus when developing treatments and designing clinical trials for FXS? There is an increased appreciation in the field of the importance of involving patients and their families in creating priorities for treatments. Weber and colleagues [20] created a survey for family members/caretakers as well as patients to identify such priorities. They found that learning difficulties, anxiety and behavioral problems are major concern areas that should be taken into consideration when developing treatments for FXS. They also point out that when designing clinical trials for treating these, or any other, symptoms there is a need to take into consideration both the age and the gender of patients to better target the treatment and assess outcomes.

Bartholomay et al. [21] expand on the differences between genders and draw attention to the fact that the scientific community mainly focused their research and clinical interventions on affected males, as females tend to exhibit milder symptoms of FXS. In this review, they present preliminary data from their prospective longitudinal study to identify factors that contribute to the overall functional outcomes in girls with FXS and may represent potential targets of intervention in this patient group.

The study by Bartholomay et al. [21] emphasizes the fact that we still have much to do to understand all the deficits in this multi-symptomatic syndrome. Language and communication skills are known to be significantly delayed in patients with FXS. However, very little work in the field has focused on language development and abilities during infancy and toddlerhood, due to the difficulties in assessing these early on in development. Reisinger et al. [22] describe their pilot study for the evaluation of early language development using the LENA (Language ENvironment Analysis) automated vocal analysis system. Consistent with previous literature, they found that caregivers of infants with FXS vocalize less around their children when compared to those of typically developing matched controls. Because language acquisition and cognitive development have been found to correlate with the amount of language in a child’s environment, this might indicate that a simple and effective intervention such as an increase in the FXS children’s caregiver’s verbal responses may positively affect the child’s language development.

Deficits in executive functions, cognitive abilities that support adaptive goal-directed behavior, are also a characteristic of FXS. In this special issue, Schmitt et al. [23] present a literature summary of executive function deficits in FXS patients. Given that deficits in executive functions have negative effects on FXS patients as well as their families, the authors emphasize the importance of better understanding the underlying affected processes and the identification of good outcome measures to develop treatments for improving these functions in FXS patients.

Another important factor when developing treatments is the time of intervention. It is believed that in a neurodevelopmental disease such as FXS, early intervention is critical for achieving the maximal therapeutic effect. The work by Reisinger et al. [22] described above, addresses language and communication skills as one example for the potential benefits of early intervention. A major confounding factor for early intervention is the age of disease diagnosis. Currently, the average age of diagnosis of FXS is three years, and usually later in affected females, which delays the onset of treatments. Okoniewski et al. [24] address this major concern and discuss screening approaches such as voluntary newborn screening (NBS) that can enable early diagnosis. They describe the creation of one such expanded NBS, called Early Check, for FXS in North Carolina. The potential benefits of such screening programs extend beyond the ability for early intervention, such as the ability of long-term follow-up and the collection of natural history data that can be used to better understand the development of the disease and its risk factors. This is especially important for documenting the relative risk of developmental differences and the identification of biological or environmental predictors of worse outcomes in infants with a premutation allele.

A major challenge for finding better drugs for FXS is the ability to translate the preclinical studies into successful clinical trials. To address this issue and provide guidelines to enhance the success of clinical trials, the Clinical Trials Committee (CTC) was formed in 2015. The CTC is made up of FXS experts as well as family stakeholders as a one-stop point of contact to consult and give input on any interventional trials planned for FXS patients. In this special issue, members of the CTC describe the changes that need to be implemented and factors to be considered when designing future clinical trials [25].

For optimal outcomes, changes should also be implemented in drug and clinical trial designs carried out within the pharmaceutical industry, a major driver of drug development. Lee et al. [26] discuss drug development from the industry point of view, and describe the modifications taking place within the industry to improve clinical trials. One of the major changes is the involvement of FXS families and patients and the consideration of their insights when designing trials. This can have a positive effect not only on the outcomes but also in the recruitment and engagement of patients in clinical trials. This, together with reaching out to the scientific community (such as the CTC), the use of better outcome measures, reducing potential placebo responses and considering the heterogeneity of the condition can all lead to the development of drugs that have a meaningful impact on FXS patients’ lives.

We hope that addressing the issues raised in this special issue will result in new studies that will help fill the knowledge gaps and identify objective outcome measures for successful clinical trials. This will ultimately advance the field in its search for effective treatments, or even a cure for FXS.
